# Rehabilitation for Children With Dystonic Cerebral Palsy Using Haptic Feedback in Virtual Reality: Protocol for a Randomized Controlled Trial

**DOI:** 10.2196/11470

**Published:** 2019-01-14

**Authors:** Reika Nicole McNish, Pramod Chembrammel, Nathaniel Christopher Speidel, Julian Jwchun Lin, Citlali López-Ortiz

**Affiliations:** 1 Department of Kinesiology and Community Health, Neuroscience Program, Beckman Institute for Advanced Science and Technology University of Illinois at Urbana-Chamapign Urbana, IL United States; 2 Health Care Engineering Systems Center College of Engineering University of Illinois at Urbana-Champaign Urbana, IL United States; 3 Department of Mechanical Engineering University of Illinois at Urbana-Champaign Urbana, IL United States; 4 Children's Hospital of Illinois OSF Saint Francis Medical Center OSF Illinois Neurological Institute Peoria, IL United States; 5 Department of Kinesiology and Community Health, Department of Dance, Neuroscience Program, Illinois Informatics Institute, Beckman Institute for Advanced Science and Technology University of Illinois at Urbana-Champaign Urbana, IL United States

**Keywords:** cerebral palsy, child, dystonia, motor skills, muscle spasticity, randomized controlled trial, rehabilitation, robotics, sensory feedback, virtual reality

## Abstract

**Background:**

Cerebral palsy (CP) is the most common developmental motor disorder in children. Individuals with CP demonstrate abnormal muscle tone and motor control. Within the population of children with CP, between 4% and 17% present dystonic symptoms that may manifest as large errors in movement tasks, high variability in movement trajectories, and undesired movements at rest. These symptoms of dystonia typically worsen with physical intervention exercises.

**Objective:**

The aim of this study is to establish the effect of haptic feedback in a virtual reality (VR) game intervention on movement outcomes of children with dystonic CP.

**Methods:**

The protocol describes a randomized controlled trial that uses a VR game-based intervention incorporating fully automated robotic haptic feedback. The study consists of face-to-face assessments of movement before, after, and 1 month following the completion of the 6-session game-based intervention. Children with dystonic CP, aged between 7 and 17 years, will be recruited for this study through posted fliers and laboratory websites along with a group of typically developing (TD) children in the same age range. We anticipate to recruit a total of 68 participants, 34 each with CP and TD. Both groups of children will be randomly allocated into an intervention or control group using a blocked randomization method. The primary outcome measure will be the smoothness index of the interaction force with the robot and of the accelerometry signals of sensors placed on the upper limb segments. Secondary outcomes include a battery of clinical tests and a quantitative measure of spasticity. Assessors administering clinical measures will be blinded. All sessions will be administered on-site by research personnel.

**Results:**

The trial has not started and is pending local institutional review board approval.

**Conclusions:**

Movement outcomes will be examined for changes in muscle activation and clinical measures in children with dystonic CP and TD children. Paired *t* tests will be conducted on movement outcomes for both groups of children independently. Positive and negative results will be reported and addressed.

**Trial Registration:**

ClinicalTrials.gov NCT03744884; https://clinicaltrials.gov/ct2/show/NCT03744884 (Archived by WebCite at http://www.webcitation.org/74RSvmbZP)

**International Registered Report Identifier (IRRID):**

PRR1-10.2196/11470

## Introduction

### Background

Cerebral palsy (CP) is the most common developmental motor disorder in children, present in 3.6 of every 1000 live births in the United States [1]. Individuals with CP demonstrate abnormal muscle tone and motor control that contribute to impaired postural control and movement coordination that compromise health [2,3]. CP is considered a static encephalopathy; there is no known cure, and the physical impairments present at early age tend to worsen with time [4].

Between 4% and 17% of children with CP present dystonic symptoms as the predominant motor impairment [5]. Dystonia is typically characterized by involuntary muscle activity that may be sustained or intermittent, thereby causing abnormal postures or movements that can be described as trembling, writhing, or contouring [6-8]. These abnormal movements are often present during attempted voluntary movement and can worsen when this movement is sustained [7].

Approximately 70% of children with CP present spastic symptoms. Spasticity manifests as (1) increased resistance to external muscle stretch that varies with flexion or extension and/or (2) resistance that quickly rises past a certain joint angle position or velocity [6,7]. Dystonia and spasticity often manifest in the same child with CP [5,7,9].

The main goals of rehabilitation in CP include promoting function while decreasing the risk for skeletal deformity and muscular atrophy later in life [10-14]. Rehabilitative therapies for CP most commonly include orthopedic surgery, botulinum toxin injections, and physical therapy (PT). PT interventions consist of intensive stretching and strengthening exercises and, more recently, high dosage robotic training [10-14]. However, dystonia worsens with PT and is often “refractory to treatment” [15]. Management of dystonia typically involves oral medication that produces little improvement and may have adverse side effects [16]. It has been recently shown that deep brain stimulation provides only moderate benefits in some patients with dystonia and none in others [16-21]. In individuals with combined spasticity and dystonia, physical interventions that improve spastic symptoms cause worsening of dystonic symptoms [7].

This worsening of dystonic symptoms is thought to be caused by abnormal signal-dependent noise modulated by the central nervous system [15]. With this in mind, we consider a low-magnitude (30% ± 10% maximum voluntary contraction) isometric force task intervention to limit signal-dependent noise that is characteristic of dystonia while minimizing movement- related sensory feedback. In addition, the intervention is designed as a virtual reality (VR) game aiming to stimulate motivation and attention as part of the rehabilitation strategy. Motivation and attention have been recognized by the National Institutes of Health Taskforce on Neuroplasticity for Clinical Applications as key for neuroplasticity during rehabilitation [8].

We hypothesize that training children with dystonic CP to match force direction targets at low magnitudes of isometric force with real-time feedback in a low dimensional space rendered in VR will improve quantitative and clinical movement outcome measures.

### Choice of Comparator

Children aged 7 to 17 years with dystonic CP and a group of typically developing (TD) children in the same age range will be recruited for the study. The CP and TD groups will be randomly assigned to an intervention or no-intervention (control) group. The control groups will be assessed in the same schedule as the intervention groups. Participants will continue with their regular PT schedule and continue their typical exercise regimen as applicable.

### Trial Design

We will conduct paired *t* tests on movement outcomes for the CP and TD groups independently. In case of non-normality, nonparametric techniques will be used. Differences before and after as well as before and follow-up will be obtained on the primary outcome measure: smoothness of movement. Differences will also be calculated on secondary outcomes for exploratory purposes. The primary outcome measure will be the smoothness index of the accelerometry signals of sensors placed on the upper limb segments. Secondary outcomes include the interaction force with the robot and a battery of clinical tests and a quantitative measure of spasticity. The study is powered for the main outcome. The desired allocation will be 1:1, with 1 participant in the control group for every participant in the intervention group for both CP and TD participants.

In the application presented here, the VR environment follows a prescribed remapping based on principal component analysis of force data generated from people with typical movement control [22,23]. Participants will, therefore, be asked to produce efforts in directions of high variance that are associated with performance of typically developed adults.

This intervention will not be incorporated into a broader health care program at this time.

### Objective

The primary objective was to establish the effect of a VR game-based force-direction training intervention on movement outcomes of children with dystonic CP using robotic feedback.

## Methods

### Study Setting

All procedures will take place at 2 sites: the Neuroscience of Dance in Health and Disability laboratory located at the University of Illinois at Urbana-Champaign (UIUC) or the Children’s Hospital of Illinois/OSF Saint Francis Medical Center. Both sites are located in Illinois, United States and are in the urban counties of Champaign and Peoria, respectively. The ethno-racial composition of the state of Illinois for the population younger than 18 years is approximately 66% white, 24% Hispanic, 16% black, 13% other, and 5% Asian. Expected total recruitment is 68 participants (Table 1) [24].

### Eligibility Criteria

The inclusion criteria are shown in Textbox 1. The exclusion criteria included not meeting all inclusion criteria.

### Interventions

All potential participants will be assessed to verify that inclusion and exclusion criteria are met during baseline assessments before being allocated into an intervention or control group. Participants in the control group will be assessed at a study site approximately 6 times, each lasting a maximum of 1.5 hours. The assessments will take place over the course of 6 to 7 weeks; there will be baseline assessments, postassessments, and 1-month follow-up assessments. Participants will attend a total of approximately 9 hours for the assessments involved in the study. All participants will be asked to continue typical PT or exercise routines outside of the study. A parent or guardian will be requested to stay in the research room at all times to further ensure participant safety and comfort.

Participants randomly allocated into the game-like intervention will participate in 6 training sessions, of up to 1 hour in duration, in addition to assessments for a total of 12 sessions. The intervention will occur over a 1- to 2-week period depending on schedule accommodations. Total participation time for intervention groups is estimated at 15 hours. Participants will play the VR game in which they will produce isometric efforts against the robot-force transducer unit that is programmed to resist their effort with a static torque control mode. The active game play time will be anywhere between 10 and 30 min depending on user preference. The source code and VR game will be available upon request.

**Table 1:** Allocation by performance site. It is expected that most participants will be tested at the Peoria location.

Performance site	Male (n=34), n (%)	Female (n=34), n (%)	Total (N=68), n (%)
Neuroscience of Dance in Health and Disability Laboratory, Kinesiology and Community Health, and UIUC^a^	12 (35)	12 (35)	24 (35)
Children’s Hospital of Illinois/OSF Saint Francis Medical Center, Peoria, Illinois	22 (65)	22 (65)	44 (65)

^a^UIUC: University of Illinois at Urbana-Champaign.

**Inclusion criteria.**

1. Aged between 7 and 17 years
2. Diagnosis with dystonic cerebral palsy (CP), for participants with CP, or have no neuromuscular conditions, for typically developing participants
3. Mild to no difficulty understanding conversations compared with others of the same age
4. Communicate age appropriately or with some difficulty, but a new listener can understand
5. No uncorrected vision
6. Hearing without the need of a hearing aid
7. No other neural, neuromuscular, or musculoskeletal conditions
8. No history of surgical procedures within 6 months before enrollment in the study
9. Participation in stable school and/or private physical or occupational therapy with a frequency no greater than 2 sessions per week for cerebral palsy groups
10. No changes in medication for the 6 months before enrollment in the study
11. Medically stable
12. No other concurrent illness
13. Received no Botox treatment within 3 months previous to the initiation of the study
14. No use of cardiac pacemakers, hearing aids, or another electronic implanted device
15. Absence of allergy to silver or skin adhesives
16. No history of seizures
17. Manual Ability Classification System score I-III

**Figure 1:**
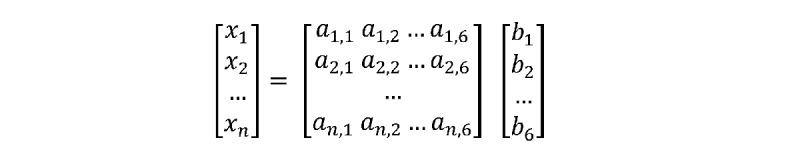
Projection into virtual reality (VR) space. The vector x in VR space is the projection of the signal vector b of n=6 force and torque signals, by the principal components matrix A.

The robot-force transducer unit consists of a 6-dimensional force and torque transducer (ATI-Nano 25, ATI Industrial Automation, North Carolina) mounted onto the end-effector of a 5 degree of freedom robotic arm (KUKA Roboter gmbh, Augsburg, Germany) that is fixed to a table. The end of the robotic arm is positioned in 5 points within the reach space of the upper limb in a vertical plane by custom software (Microsoft Visual Studio Professional 2015, Redmond, Washington). Participants will apply force to the sensor via a comfortable gripper and will receive real-time feedback on the forces and torques exerted by the hand against the robot. Feedback will be mapped onto a space displayed on a flat monitor (refresh rate: 60 Hz) or VR head mounted display (Oculus Rift ConsumerVersion1, refresh rate: 90 Hz, Oculus, Menlo Park, California).

The force and torque signals will be remapped into the VR game using the principal component analysis matrix of the average set forces and torques generated against the robot from pre-existing data of healthy adults, such that if b is the vector of n=6 force and torque signals and A is the principal components matrix then x is the projection in the VR space as shown in Figure 1. In the simplest level of the game, only the first row of the principal components matrix (n=1) is projected into the game. Following levels increase in difficulty as rows of the matrix are incorporated in the mapping (n=2, 3, …, 6).

Participants will be allowed to rest as desired between efforts that will be at 30% ± 10% of the maximal voluntary contraction for each participant. The custom game (Unity, Unity Technologies SF, San Francisco, California) requires the participants to match remapped lower-dimensional force targets that are displayed as ships in a space exploration game. A total of 14 force and torque coordinates will need to be matched, 5 times, at the 5 robot positions, in spaces of reduced dimensions ranging from 1 to 6. The game increases in difficulty (number of matching dimensions) as the participant matches the targets successfully. Participants may choose to use a screen or VR headset to play the game according to their preference. VR sessions will be limited to 30 min for each session; the remainder of the hour will be used for setup purposes and administration of the maximum voluntary muscle contractions if surface electromyography (sEMG) will be used during the session. Muscle activity will be recorded with up to 16 wireless sEMG sensors (Trigno, Delsys, Massachusetts) that are placed bilaterally on the following muscles: middle deltoid, pectoralis major, anterior deltoid, latissimus dorsi, biceps brachii, triceps brachii lateralis, flexor carpi radialis, and extensor carpi radialis.

Participants will be able to stop participating at any time without consequence. If a child has a first seizure during the study, the inclusion and exclusion criteria will no longer be met, and participation in the study will be terminated. A procedural checklist will be followed during experiments.

### Outcomes

All participants will be assessed for outcome measures at a site using sEMG sensors embedded with inertial measurement units (ATI-Nano 25, ATI Industrial Automation, North Carolina) and the robot-force transducer unit. All assessments and measurements are noninvasive and involve minimum risk to the participants. The accelerometry data will be used to assess smoothness of movement during the execution of a prescribed movement and measures muscle activation patterns during assessments involving the robot-force transducer unit used to measure force and torque outputs within the reachable space of the upper limbs of the participant. In addition, the robot is programmed to produce a path of zero resistance, within the reachable space, that provides haptic feedback perpendicular to the path. For assessing children with CP, we will collect clinical and quantitative measures of upper limb range of motion, motor function, dystonia, and spasticity. The outcome measures will be obtained before, after the intervention, and at 1-month follow-up (Textbox 2).

Assessments 1 to 4 are standard clinical questionnaires and tests. Assessment 5 is a quantitative test of spasticity based on sEMG recordings and angle velocity of a joint by manual manipulation.

Assessment 6 involves the robot, force sensor, and sEMG. A predetermined zero-force path has been programmed by the research team to allow the robot to move through straight lines connected to 5 points on a vertical plane. These positions coincide with the 5 positions of the training intervention, resting in a reachable rhombus-like configuration. The participants will attempt to find and move along a zero-force path. The force transducer will measure the forces exerted by the participant as they do so. This task will be limited to a 7-min period or until the task is complete.

**Measured outcomes. Measures for all participants (ie, listed from 6 to 10 will be done for all participants). The remaining articles are outcome measures for participants with cerebral palsy, that is, these will be specific to participants with cerebral palsy.**

1. Dyskinesia Impairment Scale
2. Selective Control of the Upper Extremity Scale
3. Quality of Upper Extremity Skills Test
4. Tardieu Test
5. Montreal Spasticity Rating Test
6. Zero-Force Channel Assessment
7. Surface electromyography and accelerometry
8. Forces and torques against force sensor
9. World Health Organization Disability Assessment Schedule 2 Children and Youth
10. Qualitative Feedback Module

**Figure 2:**
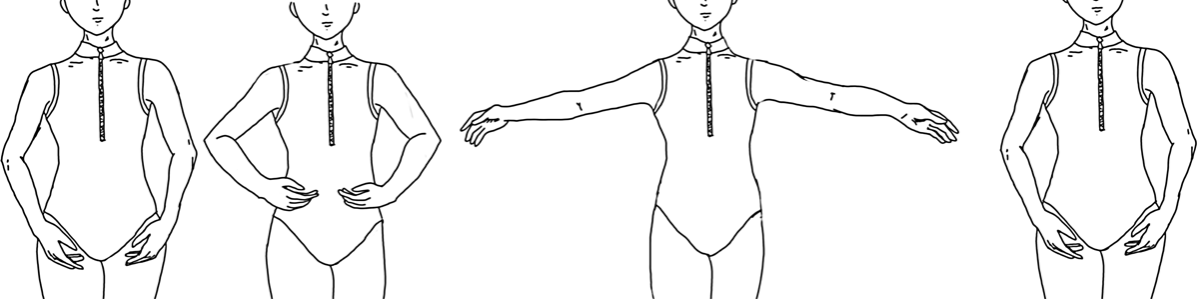
Royal Academy of Dance first port de bras. From left to right: en bas, first position, second position, and en bas. Participants will be asked to move through these positions during assessment 7 to measure muscle activity patterns and smoothness of movement.

Assessment 7 will be used as necessary during assessments and on the first and last day of gameplay. Accelerometry and sEMG data will be collected during the execution of a first *port de bras*. The first *port de bras* will follow the format from the Royal Academy of Dance as demonstrated (Figure 2). Accelerometry data will be integrated to calculate the smoothness index on the velocity profile of the trajectories [25]. Data will be analyzed for changes in muscle activity patterns and smoothness of movement. The sEMG data will also be acquired for maximum voluntary efforts.

Assessment 8 measures force and torque inputs using the force and torque sensor that is mounted on the end-effector of the robot. A maximum voluntary effort will be made by the participant’s dominant arm; the sensor will be mounted to a sturdy table for this task. The subject will be asked to push and pull along cardinal directions to determine the maximum force output that will be used to customize sensitivity subsequent efforts for each participant. Participants will also be assessed with the force sensor in 14 different directions at 30% ± 10% of the maximal force.

Assessment 9 is the World Health Organization Disability Assessment Schedule II-Children and Youth (WHODAS II-CY) that characterizes the children’s level of disability. This measure will only be used for population demographic purposes.

Assessment 10 will be the qualitative feedback module, which will provide qualitative feedback on participants’ experiences.

### Participant Timeline

The full study timeline is approximated to take 20 weeks (Table 2). Possible schedules for participation will vary per participant; however, assessments will be within ± 1 week from the suggested dates for accommodations (Tables 3 and 4).

### Sample Size

No previous sample data exist for this type of study that would enable sample size calculations. Data from a study on finger muscle control in children with dystonic CP with a mean difference effect size for within-subjects designs of dz=2.98, alpha=.05, and power=0.8 yields a required sample size of n=4 in each comparison group [26]. Given the wide age range and motor impairment characteristics in the eligibility criteria for this study, we propose a sample size of n=17 with a total number of 68 participants. We expect a 25% attrition rate that will approximately yield a total of 13 participants per group. We consider that this number is a conservative estimate for a randomized controlled trial in the pilot phase.

### Recruitment

The participants will be neither students nor employees of the research team personnel. We intend to recruit participants from the Central Illinois community. Physicians involved in the experiment from the Children’s Hospital of Illinois/OSF Saint Francis Medical Center will assist in referring participants and may distribute flyers with contact information regarding the study. In addition, participants will be recruited through posted flyers, advertisement in the Daily Illini, E-week, local newspapers, and laboratory websites. Once participants’ parent or guardian receives information about the study, they will have the option to contact the principal investigator as indicated in the study information flyer. We are not accessing patient records for recruitment or Illinois schools. The final decision on inclusion will be made by the principal investigator in accordance to the inclusion and exclusion criteria of the experiment.

**Figure 3:**
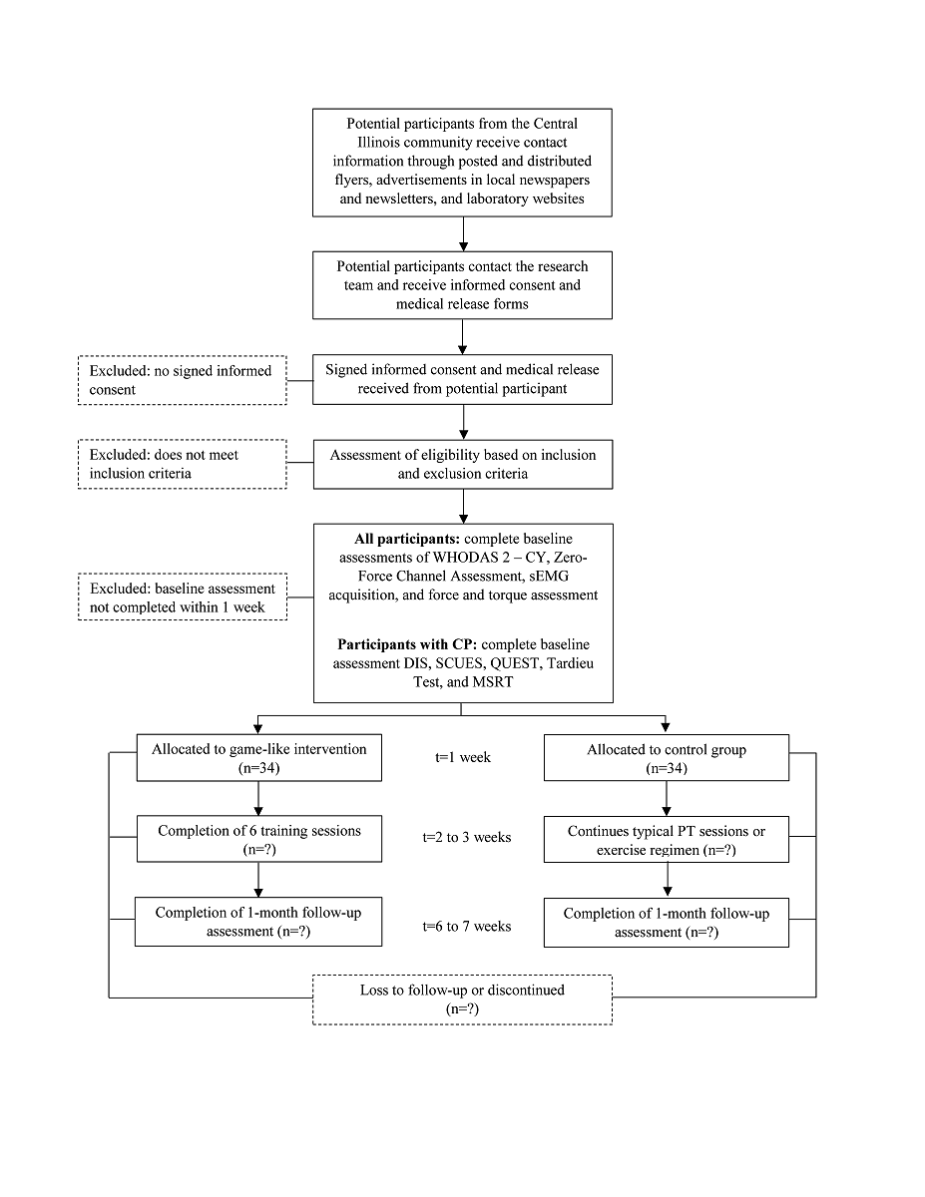
Standard Protocol Items: Recommendations for Interventional Trials (SPIRIT) flow diagram of participants. CP: cerebral palsy; DIS: Dyskinesia Impairment Scale; MSRT: Montreal Spasticity Rating Test; PT: physical therapy; QUEST: Quality of Upper Extremity Skills Test; sEMG: surface electromyography; SCUES: Selective Control of the Upper Extremity Scale; WHODAS 2-CY:World Health Organization Disability Assessment Schedule II-Children and Youth.

During the initial contact interview, research assistants will read a script describing the study and, if interested, parents or guardians will be provided with the participant medical form, to be completed by the parent or guardian and physician, and the consent form. A model consent form is provided in the Multimedia Appendix 1. Screening materials will be kept for participants that enroll in the study and destroyed for those that do not meet the criteria or decide not to enroll. A schematic diagram is included (Figure 3).

**Table 2:** Full study timeline. Experiment tasks are divided into weeks.

Time (weeks)	1	2	3	4	5	6	7	8	9 to 14	15 to 20
Recruitment and enrollment	✓^a^	✓	—^b^	—	—	—	—	—	—	—
Baseline assessments	✓	✓	—	—	—	—	—	—	—	—
Intervention period	—	✓	✓	—	—	—	—	—	—	—
Postassessments	—	✓	✓	—	—	—	—	—	—	—
1-month follow-up	—	—	—	—	—	✓	✓	—	—	—
Data processing	—	—	✓	✓	✓	✓	✓	✓	✓	—
Data analysis	—	—	✓	✓	✓	✓	✓	✓	✓	✓

^a^✓ corresponds to times when activity occurs.

^b^Empty cells indicate times when activity is not expected to occur.

**Table 3:** Possible schedule 1. Interventions in this schedule will take place over a period of 2 weeks with days between. Assessments will take place over 2 days. This schedule is subject to change based on the participants’ availability.

Time (weeks)	1	2	3	4	5	6	7
Saturday	Assess	—^a^	Assess	—	—	—	Assess
Sunday	Assess	—	Assess	—	—	—	—
Monday	Game	Game	—	—	—	—	—
Tuesday	—	—	—	—	—	—	—
Wednesday	Game	Game	—	—	—	—	—
Thursday	—	—	—	—	—	—	—
Friday	Game	Game	—	—	—	Assess	—

^a^Empty cells indicate no activity.

**Table 4:** Possible schedule 2. Interventions in this schedule will have no days between sessions. Assessments will take place over 2 days. This schedule is subject to change based on the participants’ availability.

Time (weeks)	1	2	3	4	5	6	7
Saturday	Assess	Game	—^a^	—	—	Assess	—
Sunday	Assess	Assess	—	—	—	Assess	—
Monday	Game	Assess	—	—	—	—	—
Tuesday	Game	—	—	—	—	—	—
Wednesday	Game	—	—	—	—	—	—
Thursday	Game	—	—	—	—	—	—
Friday	Game	—	—	—	—	—	—

^a^Empty cells indicate no activity.

**Table 5:** Participant group allocation. All participants will be divided into intervention and control groups.

Participants	Intervention (n=34), n (%)	No intervention (control; n=34), n (%)	Total (N=68), n (%)
Children with cerebral palsy	17 (50)	17 (50)	34 (50)
Typically developing children	17 (50)	17 (50)	34 (50)

### Allocation

After the initial contact interview, assessment of inclusion and exclusion criteria, and completion of the consent and assent forms, the participant will be allocated to a control or intervention group using a blocked randomization method (Table 5). The block sizes and randomized sequences will be hidden from those who enroll or allocate participants to prevent predictability of the next assignment. Allocation will be concealed by using sequentially numbered, opaque, sealed envelopes. Different members of the research team will allocate the sequence, enroll participants, and assign them to groups.

### Blinding

Research team members administering clinical assessments will be blinded from participant allocation. Participant’s allocation will not be revealed during the course of the study.

### Data Collection Methods

All assessors are trained to conduct the assessments for children with and without CP. Outcome data will be collected at assessments.

The Dyskinesia Impairment Scale measures the severity of dystonia and choreoathetosis when an individual is at rest or conducting movement. It was found to show good to excellent reliability and validity [27]. It is also notable that in a 2012 systematic review of measures of dystonia and choreoathetosis, this scale is listed as the only clinical tool that examines choreoathetosis and dystonia in the same scale [28].

The Selective Control of the Upper Extremity Scale is a video-based tool to measure selective control of upper limb tasks. Psychometric analysis shows “comparable validity to other accepted video-based clinical assessment tools for the upper extremity in children with CP” with content validity ratio values indicating substantial agreement for most items [29].

The Quality of Upper Extremity Skills Test is 36 items in length and measures upper limb movement, hand function, and cooperativeness in children with CP. It has been found to be reliable to assess children with CP in the age range of 18 months and 8 years, with increased reliability in children aged up to 12 years [30]. It has also been found to show adequate to excellent validity [31,32].

The Tardieu scale examines spasticity with quantified measures of the responses to stretch reflexes of discrete velocity. This scale shows high test retest and poor to moderate inter-rater reliability; the Tardieu scale performs better than other similar measures, however, indicating it may be more reliable [33].

The Montreal Spasticity Rating Test is a quantitative measure of spasticity that identifies resistance to external forces of stretch tasks. This test identifies the point at which the stretch reflex is activated in a muscle. Documentation on the reliability and validity of this test is unavailable.

The WHODAS II-CY is a self-administered 36-item document that assesses daily issues surrounding health conditions such as illnesses, injuries, and problems with mental health. In a validity study, it was found to show good reliability; however, limitations regarding options for those without significant disabilities were present [34].

Plans to promote participant retention include payment at the end of, or separation from, the study.

### Data Management

All data will be deidentified. Paper medical records will be brought by participants to the testing site or sent by US mail to the principal investigator’s university address; they will be stored under double lock. All consent and assent forms will be completed at a testing site. Clinical test results performed during the experiment will be paper-recorded, and deidentified data will be inputted electronically for data analysis. Input of electronic records will be verified by 2 different members of the research team. Tests that record data electronically, such as sEMG, will be kept electronically. Data collected from source documents will be inputted into an encrypted and password-protected computer that is secured by the campus firewalls.

Paper records will be locked in a double-locked cabinet. All electronic data will be stored on password-protected computer that is secured by the campus firewalls. The computer designated for data collection and experimentation will not be connected to the internet for heightened security. All deidentified data will be submitted to an online repository as required for publication of randomized clinical trials.

### Statistical Methods

The assessment and training protocols target improvements in selective motor control and amelioration of dystonia. We will conduct paired *t* tests on movement primary and secondary outcomes for the CP and TD groups independently. In case of non-normality, nonparametric techniques will be used. Differences before and after as well as before and follow-up will be obtained on the primary outcome measure: smoothness of movement. Tests on secondary outcomes are for exploratory purposes only. Bonferroni corrections will be applied as needed.

Missing values will be omitted from calculations or corrected for according to standard statistical techniques.

### Monitoring

#### Data Monitoring

A data monitoring committee will not be needed as this will be a minimal risk trial.

#### Risks Related to the Intervention

Physical discomfort caused by the weight of the VR headset or simulation sickness may arise with extended VR use. Muscle soreness because of repetitive use of muscle groups may occur, and skin irritation from adhesives is possible. There is a risk of seizure using the VR headset estimated to be 1 in 4000 in the general population.

#### Safety Measures

To avoid simulation sickness, the programed VR images are slow, soothing, and were created following Oculus Best Practices Version 310-30000-02 as provided by Oculus VR, LLC. In addition, any health risks associated with VR headset use in children will be mitigated by referencing the Health and Safety Warnings provided by Oculus VR, LLC; children younger than 13 years will not be permitted to use the virtual headset. The participant has the option to play the game on a flat monitor or using a VR headset according to their preference. If simulation sickness arises, no medication will be administered as it is not a severe side effect of VR. Grounding exercises may be done if needed or the participant may decide to stop for the day or continue without the headset. Headset use will be limited to 30-min intervals.

Aside from maximum voluntary efforts, most efforts that participants produce will be 30% ± 10% of their maximum voluntary effort. The experiment will also be conducted in a seated position, reducing risk of injury because of falls, and ample care will be taken to ensure the participants’ comfort as needed including provision of seat cushions. Trained research personnel will use gait belts, when needed, to transport participants from their wheelchair to the chair used for the experiment and back to their wheelchair as necessary. If their wheelchair allows for interaction with the robot, no transfer will take place. Surfaces that come in contact with participants are wiped down with hospital grade antiviral and antibacterial wipes before and after use.

Potential participants with a history of seizures will not be included in the study. Research personnel are trained to manage the rare event of a first seizure and follow the guidelines of the British Epilepsy Association: (1) remove objects nearby and cushion the head, (2) note the time when jerking starts, (3) place the person on one side of their body in recovery position after any jerking stops, and (4) stay with the person. In addition, (5) movement will not be restrained, (6) no objects will be put in the mouth, (7) the person will not be moved, and (8) no food or drink will be given until full recovery [35]. If the person is seated in a wheelchair, the brakes will be put on, and the person will be gently supported to prevent falling out of the chair if necessary. Research personnel will call for an ambulance, as it would be the person’s first seizure. The experiment will be stopped, and the participants’ guardian will be advised to seek immediate medical attention. The guardian will be asked to stay present during each session. If a seizure were to occur, the participant will not be allowed to continue with the study as the inclusion and exclusion criteria will no longer be met.

The robot (KUKA Roboter gmbh, Augsburg, Germany) is approved for human-robot collaborative mode and is assured to perform as instructed. A trained researcher will be next to the participant and robot at all times and will be prepared to unplug the robot if any unexpected movement of the robot occurs. The participant is not secured to the robot as well, which allows the participant to release the end effector if necessary. In addition, if a large force is applied to the robot, the uppermost link will turn off. The gripper mounted on the sensing surface of the force and torque transducer is rounded with no sharp edges.

Additional safeguards are included in the inclusion and exclusion criteria, in the consent and assent forms, and in the protocol design. In addition, the assent forms have extensive language describing the study and allow for the termination of participation by the participant at any time and for any reason.

We have tested the system extensively and do not expect any major changes or bugs. In case of any unexpected event, including situations of power failure in which testing has proven to cause no harm to the participant, the experiment will be stopped, and the research team will test the system to ensure functionality. Participants will be asked to return to the site on another day and will be reimbursed for the additional time.

#### Benefits

The participants may not receive any direct benefit beyond the satisfaction of participating in research, playing a VR game, and advancing the knowledge of motor control and coordination in humans. However, participants may notice improved control of movement of the upper limbs. The benefit to society rests on the advancement of our understanding of motor control and coordination in humans as well as improving diagnostic specificity in CP and providing a possible therapy modality. This information may be used to train future physicians.

#### Harms

All communications will be sent to both the Peoria and UIUC institutional review boards (IRBs).

#### Auditing

Auditing will be done as per the policies of the sponsor and the bodies that have sponsor oversight.

#### Ethics and Dissemination

Approval from the study sites institutional review boards will be sought. All protocol modifications will be communicated to both IRBs.

Potential participants and a parent or guardian will have the option to sign an assent and consent form, respectively, for participation in the study.

Identifiable elements including names, phone numbers, street addresses, city or state, zip code, email addresses, date of birth, grade level in school, and photos and videos (no close-up footage) will be collected. Screening materials will be kept for the participants that enroll in the study and destroyed for those that do not meet the criteria or decide not to enroll. Authorization for use and disclosure of the participants’ personal health information for this specific study does not expire. The data will be kept for 5 years after publication, as required by the American Psychological Association. Identifiers will be destroyed 5 years after the completion of the study.

Personal contact information will be used for the study team to contact participants during the study. Health information and results of tests and procedures are being collected as part of this research study for fulfillment of inclusion criteria and for the advancement of clinical care. By signing assent and consent forms, permission is given to the principal investigator and the research team to use the protected health information for the purposes of the study. Permission is also granted to the OSF Healthcare System and the University of Illinois College of Medicine at Peoria to disclose or release the participants’ protected health information for this study.

If suspected abuse, neglect, or exploitation of a child or a disabled or elderly adult is disclosed, the researcher or members of the study staff will report the information to Child Protective Services, Adult Protective Services, or a law enforcement agency.

Images and videos will be stored without personal identifiers associated with the files apart from the image or video itself. If permission is given, these photos could be included in scholarly publications in print and electronic form, which will allow participants’ faces to potentially be visible and recognizable by anyone reading the publications. Photos and videos may also be presented at meetings or conferences without any personal identifiers attached to the photos and videos other than the content itself. Data will be kept with coding and will only be viewable by lab personnel who are associated directly with the maintenance of data for this study.

Video footage with audio will be recorded at all sessions to ensure safety and adhesion to study protocols as well as to record the study. The footage is being taken to ensure the rights of participants and for the researchers alike. By signing the consent and assent forms, authorization is given for the principal investigator and research team to record the participant during participation of the study and to share the footage with the following items if emergencies arise or the research protocol is not properly followed:

- The IRBs
- The Office of Human Research Oversight
- Authorized members of the University of Illinois College of Medicine Peoria workforce
- Representatives of the university committee and office that reviews and approves research studies
- Office for Protection of Research Subjects
- Other representatives of the state and university responsible for ethical, regulatory, or financial oversight of research
- Federal government regulatory agencies such as the Office of Human Research Protections in the Department of Health and Human Services

## Results

The trial has not started and is in queue for local IRB approval. We expect end results to be available by May 2019.

## Discussion

### Research Team

The members of the research team have diverse backgrounds: kinesiology, neuroscience, pediatric surgery, mechanical and electrical engineering, game design and development, and dance. This allows for a novel rehabilitation paradigm incorporating haptic feedback in VR targeting dystonia. In a routine application setting, similar levels of human involvement may be necessary to run the training sessions.

### Limitations

A limitation of this study may be the limited intervention timeline. A total of 6 sessions may not be enough time for changes to occur; however, a similar study for the treatment of spasticity did demonstrate improvements in this timeline [23]. The small sample size and limited geographic area preclude absolute generalization of the results. Larger clinical trials would be necessary for generalization. In addition, as a characteristic of any rehabilitation intervention trials, participant blinding to the intervention or having a true placebo group is impossible.

### Comparison With Prior Work

No prior work has been completed.

### Conclusions

This study aims to examine movement outcomes of children with and without dystonic CP after a VR rehabilitation intervention using haptic feedback. We anticipate improvements in smoothness of movement after the intervention as well as in clinical movement tests.

## Appendix

Multimedia Appendix 1 Model Consent Form.

## Abbreviations

CP: cerebral palsy
IRB: institutional review board
PT: physical therapy
sEMG: surface electromyography
TD: typically developing
UIUC: University of Illinois at Urbana-Champaign
VR: virtual reality
WHODAS II-CY: World Health Organization Disability Assessment Schedule II-Children and Youth
