# Supplementary material for: Rehabilitation for Children With Dystonic Cerebral Palsy Using Haptic Feedback in Virtual Reality: Protocol for a Randomized Controlled Trial
Source: JMIR Res Protoc. 2019 Jan 14;8(1):e11470. doi: 10.2196/11470 (PMC6682270; doi:10.2196/11470)
Supplement: Multimedia Appendix 1 [file resprot_v8i1e11470_app1.pdf]

RESEARCH SUBJECT
INFORMED CONSENT FORM

Protocol Title: Movement impairment characterization and rehabilitation for spastic/dystonic cerebral palsy using robotic haptic feedback in virtual reality

Principal Investigator: Citlali López-Ortiz, PhD, MA
[redacted]

Co-Investigators: Julian J. Lin, MD
[redacted]

Susan Caldecott-Johnson, MD
[redacted]

Emergency Contact: Citlali López-Ortiz, PhD, MA
[redacted]

Susan Caldecott-Johnson, MD
[redacted]

Funded by the Jump Arches Grant Program.

Why am I being invited to volunteer?

You are being invited to give permission for your child to participate in a research study. "Research" designates an activity designed to test a hypothesis, permit conclusions to be drawn and thereby to develop or contribute to generalizable knowledge, whereas "practice of medicine" refers to interventions designed solely to enhance the well-being of an individual patient. Research subjects may or may not benefit from research procedures. Federal regulations require that you are informed of the research you are being invited to volunteer for and your signature indicating that you have been informed about the research. You are being invited to volunteer since you meet the requirements for enrollment into this study. Your participation is voluntary which means you can choose whether or not you want to participate. Before you can make your decision, you will need to know what the study is about, the possible risks and benefits of being in this study, and what you will have to do in this study. The research team is going to talk to you about the research study, and they will give you this consent form to read. You may also decide to discuss it with your family, friends, or family doctor. You may find some of the medical language difficult to understand. Please ask the study doctor and/or the research team about this form. If you decide to participate, you will be invited to sign this form. Your signature on this form is voluntary and does not waive any of your legal rights or make any institutions or persons involved in this research any less responsible for your well-being. You are free to refuse to participate or to withdraw from the study at any time without penalty or loss of benefits to which you would otherwise be entitled.

A description of this clinical trial will be available on http://www.ClinicalTrials.gov, as required by U.S. Law. This web site will not include information that can identify you. At most, the web site will include a summary of the results. You can search this web site at any time.

Who is the Principal Investigator for this Study?

Citlali López-Ortiz, PhD, MA
[redacted]

What is the purpose of this research study?

The purpose of this study is to test a rehabilitation intervention for children with spastic/dystonic cerebral palsy using a virtual reality game. The long-term objective of this project is to assist physician training.

If initial tests and procedures show that your child can be in the study, your child will be "randomized" into one of the study groups described below. Randomization

means that you are put into a group by chance. It is like flipping a coin or pulling numbers from a hat. Neither you, your child, nor the Principal Investigator can choose what group your child will be in. Your child will have an equal chance of being placed in any group.

If you are in group 1: Your child will need to come to the study site [redacted] to participate in the study. If your child is participating in this group, your child will come a total of approximately six times. Your child will be asked to come to the assessments, when tests and procedures will be done, each lasting a maximum duration of 1.5 hours. The assessments will take place three times over the course of 6 to 7 weeks. Participation in the study will take a total of about 9 hours. A parent or guardian is requested to stay on the research site at all times if anything were to happen to the participating child.

If you are in group 2: Your child will need to come to the study site [redacted] to participate in the study. If your child is part of the intervention group, your child will come a total of approximately twelve times. Your child will be asked to come to the assessments, when tests and procedures will be done, each lasting a maximum duration of 1.5 hours. The assessments will take place three times over the course of 6 to 7 weeks. Your child will be asked to return to the study site after the first set of assessments to participate in intervention sessions. There will be six intervention sessions lasting approximately one hour in duration and will occur over a 1- to 2-week period. Participation in the study will take a total of about 15 hours. A parent or guardian is requested to stay on the research site at all times if anything were to happen to the participating child.

How long will my child be in the study?

We think you will be in this study for approximately 6 to 7 weeks.

How many other people will be in the study?

About 80 people may take part in this study. Each participant will be seen individually and not in groups.

What is involved in this study?

Below are two sample schedules. **Group 1** will be asked to come to assessment days, in bold, whereas **Group 2** will be asked to come for both the assessment and intervention days.

	Saturday	Sunday	Monday	Tuesday	Wednesday	Thursday	Friday
1	**Assessment**	**Assessment**	Intervention		Intervention		Intervention
2			Intervention		Intervention		Intervention
3	**Assessment**	**Assessment**					
4							
5							
6							**Assessment**
7	**Assessment**						

	Monday	Tuesday	Wednesday	Thursday	Friday	Saturday	Sunday
1						**Assessment**	**Assessment**
2	Intervention	Intervention	Intervention	Intervention	Intervention	Intervention	**Assessment**
3	**Assessment**						
4							
5							
6						**Assessment**	**Assessment**

Your child will need to come to the study site [redacted] about 6 to 12 times over the next 6 to 7 weeks.

Each of those visits will take about 1 to 1.5 hours, depending on the day and group your child is in.

All children with CP will continue their typical physical therapy sessions. If your child has CP and is in **Group 2**, intervention sessions will be done in addition to typical physical therapy attendance.

If your child is typically developing, he or she will be expected to continue typical exercise routines outside of the study.

The study procedures are separated into two parts: assessments and intervention sessions. During the assessments, we will ask your child to push and pull on a force sensor mounted on a robot. We will be measuring your child's muscle activity with up to sixteen wireless surface electromyography (sEMG)

electrodes on muscles of the arms and trunk. The robot is approved for human-robot collaborative mode and the sEMG sensors will be placed on the skin like stickers and should not cause pain. Questionnaires and tests will also be conducted to assess baseline demographics and movement quality. If your child is in **Group 2**, in the six intervention sessions, your child will use the same robot to play a virtual reality game on a computer. Again, we will use sEMG sensors to measure muscle activity and the force sensor on the robot will be measuring forces and torques while he/she plays the game. Children under the age of 13 will not be permitted to use the virtual reality headset due to potential health and safety risks. Children 13 years of age or older will be given the option to use the headset if they desire. The headset can be removed at any time during the study and the game can be played using a flat computer monitor.

The researchers conducting this research study would like your permission to allow your child's data to be used in the future for research purposes. By signing this document, you will be giving permission for the researchers to do so.

The data to be collected and used will consist of the raw sEMG data recording muscle activation as well as the force outputs taken by pushing on the robot. Data collected is not attached to any personal identifiers. Data acquired from assessment sessions may also be used, but will not be attached to any personal identifiers.

Future studies may provide additional information that will be helpful in understanding muscle activation patterns in people. The de-identified data will be submitted to a data repository for any type of future research. It is likely that what we learn from the future research may have no direct benefit to your child.

What about Confidentiality?

We will keep your participation in this research study confidential to the extent permitted by law. However, it is possible that other people such as those indicated below may become aware of your participation in this study and may inspect and copy records pertaining to this research. Some of these records could contain information that personally identifies you.

- Federal government regulatory agencies
- [redacted] Institutional Review Boards (the committees charged with overseeing research on human subjects)

- The Office of Human Research Oversight [redacted] (the office which monitors research studies)

- Representatives of the university committee and office that reviews and approves research studies, the Institutional Review Board (IRB) and Office for Protection of Research Subjects

- Other representatives of the state and university responsible for ethical, regulatory, or financial oversight of research

- Federal government regulatory agencies such as the Office of Human Research Protections in the Department of Health and Human Services

- If you disclose actual or suspected abuse, neglect, or exploitation of a child or a disabled or elderly adult, the researcher or members of the study staff will report the information to Child Protective Services, Adult Protective Services, and/or a law enforcement agency.

Data will be de-identified. Paper records will be locked in a double-locked cabinet in a safe location. All electronic data will be stored on [redacted] computers which are encrypted, password protected, and secured by the campus firewalls and the associated security they provide. Data stored will not have records identifying data to a subject's name or identity. Additionally, the computer designated for data collection and experimentation will not be connected to the internet for heightened security. All de-identified data will be submitted to an online repository as required for publication of randomized clinical trials. Data collected from the force sensor and surface electromyography will be submitted to a data repository as de-identified data.

If you decide to allow us to take video and still images of your child during the experiments for research and teaching purposes, the full image of your child will be stored without personal identifiers associated with the files apart from the image or video itself. These photos could be included in scholarly publications in print and/or electronic form, which will allow your child's images including your child's face could be visible and recognizable by anyone reading the publications. Photos and videos may also be presented at meetings or conferences without any personal identifiers attached to the photos and videos other than the content itself.

Samples/data will be kept with coding, meaning that all direct identifiers like your child's name will be removed from the sample and/or data and replaced with a research number. A listing of your name and associated research number will be kept separately for privacy protection and will only be viewable by lab personnel that are associated directly with the maintenance of data for this study.

What are the possible risks or discomforts?

Since this INTERVENTION is unproven, there may be unexpected or unanticipated problems that may arise during your child's participation in this study. Some risks may be currently unknown or unforeseeable. Most of the anticipated or known risks are listed below, but they will vary from person to person. This is a low risk study. You should discuss these with the research team and/or your regular physician if you are concerned. Risks related to the INTERVENTION we are studying include:

Likely

- Discomfort with extended virtual reality headset use regarding vection, an illusory sense of movement which may cause movement sickness, and physical discomfort which may be caused by the weight of the headset
    - The headset is not required for participation in the study and may be removed at any time
    - VR headset use will be limited to 30-minute intervals
- Muscle soreness due to repetitive use of muscle groups
    - Breaks may be taken as desired to rest and breaks to drink water will be allowed

Less Likely

- Skin irritation from adhesion and removal of sEMG sensors. These tapes are designed for human use and we provide lotion to minimize discomfort when removing the adhesive tapes.
- There is a risk of seizures (1 in 4000 in the general population) when using a virtual reality headset as well as when playing a video game on any screen. Since your child has never had seizures, this risk is considered to be lower.
    - Research personnel are trained to manage the rare event of a first seizure and will call for an ambulance.
    - The experiment will be stopped and you will be advised to seek immediate medical attention.
    - If a seizure were to occur, the participant will not be allowed to continue with the study as the inclusion/exclusion criteria will no longer be met.

Rare but serious

- None are known

It is important to call the researcher or your regular physician when you think you are having problems, even if they are not included on the above list.

What if new information becomes available about the study?

During the course of this study, we may find more information that could be important to you. This includes information that, once learned, might cause you to change your mind about giving permission for your child to be in the study. We will notify you as soon as possible if such information becomes available.

What are the possible benefits of the study?

There may be no direct benefit to your child if you decide to allow your child to participate in this research. The value of the INTERVENTION is unproven. A clinical trial is one of the most exact ways to test if the intervention is beneficial for children diagnosed with spastic/dystonic or dystonic cerebral palsy. It may also become possible to further understand dystonia for movement classification and clinician training. This new knowledge will benefit future patients and medical investigators. If the INTERVENTION is found to be better, you may still receive no direct benefit if you are assigned to the “control” group, instead of the INTERVENTION.

What other choices do I have if I do not allow my child to participate?

Instead of being in this study, you have these options:

- You could choose not to participate in this study

Will my child be paid for being in this study?

Your child will receive monetary compensation for involvement in the study in the amount of [redacted] per session. Both, assessment sessions and intervention sessions will be remunerated. Monetary remuneration will be issued at the completion of the study in the form of a gift card. In the case of equipment malfunction, or other extenuating circumstance that prevents the completion of a testing session, you will be asked to bring your child back and repeat the testing and he/she will receive additional payment of [redacted] for each repeated visit. If you choose to withdraw your child from the study early for any reason, the amount will be prorated at a rate of [redacted] per session. The total approximate remuneration amount will be no more than [redacted]. Participants will also be reimbursed for parking costs at the UIUC location at a rate of [redacted] per hour while participating in the study.

What are the costs for participating in this research?

There are no costs to you for participating in this research.

What happens if I am injured or hurt during the study?

If your child gets ill or injured from being in the study, you should let the principal investigator know right away. You should contact Dr. [redacted] at telephone number [redacted].

You should let any health care provider who treats your child know that you are in a research study. If you do seek medical treatment, please take a copy of this document with you because it may help the doctors where you seek treatment to treat you. It will also provide the doctors where you seek treatment with information they may need if they want to contact the research doctors.

You or your health insurance plan will be billed. No money has been set aside to pay the costs of this treatment. Health insurance plans may or may not cover costs of research-related injury or illness. You should check with your insurance company before deciding to participate in this research study. Costs not covered by insurance could be substantial. Please ask about any added costs or insurance problems.

[redacted] has not set aside any money to pay you or to pay for your child's treatment if you get ill or injured from being in the study. There are no plans for [redacted] to provide other forms of compensation (such as lost wages or pain and suffering) to you for research related illnesses or injuries.

By signing this form, you are not giving up any legal rights to seek compensation of injury.

Concerning your child's rights as a research subject, you may also contact the Peoria Institutional Review Board by calling [redacted]. You may also contact the [redacted] Office for the Protection of Research Subjects at [redacted].

When does the Study end?

Your child can stop participating at any time. However, if you decide to stop participating in this study, we encourage you to talk to the researcher and your regular physician first. If you decide to stop participating, you may still be invited to provide the researcher with information through telephone calls or clinic visits.

There are no serious consequences of withdrawing from the study.

This study is expected to end after all participants have completed all visits, and all information has been collected. This study may also be stopped at any time by your physician, the study Sponsor, or the Food and Drug Administration (FDA) without your consent because:

- The Principal Investigator feels it is necessary for your health or safety. Such an action would not require your consent, but you will be informed if such a decision is made and the reason for this decision.
- You have not followed study instructions.
- The Sponsor, the study Principal Investigator, or the Food and Drug Administration (FDA) has decided to stop the study.

Although the study is ongoing, you may discontinue participation or the principal investigator may ask you to withdraw your child from the study at any time. If you decide not to allow your child to participate, you are free to leave the study at any time without consequence.

Who can see or use my protected health information? How will my protected health information be protected?

Your child's privacy and the protection of your child's health information are important to us. This section of the consent will cover:

- What protected health information about your child will be collected in this study
- Who will use your child's information and why
- Who may disclose your child's information and to whom
- Your rights to access research information about your child
- Your right to withdraw your authorization (approval) for any future use of your child's protected health information

1. Protected health information about you that will be collected in this study

The following protected health information will be collected, used for research and may be disclosed or released during your child's involvement with this research study:

- Information provided by the Participant Medical Form including your child's name and medical history

2. Why your protected health information is being used

Your personal contact information is important for the study team to contact you during the study. Your child's health information and results of tests and procedures are being collected as part of this research study for fulfillment of inclusion criteria and for the advancement of clinical care. You are also giving OSF Healthcare System and the University of Illinois College of Medicine at Peoria permission to disclose or release your child's protected health information for this study.

3. Who may use or disclose your personal health information

By signing this document, you are giving permission to Dr. [redacted] and her research team [redacted] to use the protected health information described above for the purposes of this study and for the advancement of clinical care. You are also giving [redacted] permission to disclose or release your child's protected health information for this study.

4. Who might receive your personal health information?

As part of the study, the Principal Investigator, study team, and others listed above in item number 3 may disclose your child's protected health information, including the results of the research study tests and procedures to the following:

- [redacted] Institutional Review Boards (the committees charged with overseeing research on human subjects)
- The Office of Human Research Oversight (the office which monitors research studies)
- Authorized members of the [redacted] workforce who may need to access your information in the performance of their duties, for example: to provide treatment, to ensure integrity of the research, accounting or billing matters, etc.
- Representatives of the university committee and office that reviews and approves research studies, the Institutional Review Board (IRB) and Office for Protection of Research Subjects;
- Other representatives of the state and university responsible for ethical, regulatory, or financial oversight of research;
- Federal government regulatory agencies such as the Office of Human Research Protections in the Department of Health and Human Services

The Principal Investigator or study team will inform you if there are any changes to the list above during your child's active participation in the trial. Once information is disclosed to others outside this institution, the information may no longer be covered by the federal privacy protection regulations.

- In all disclosures outside of this institution's system, you will not be identified by name, social security number, address, telephone number, or any other direct personal identifier unless disclosure of the direct identifier is required by law.
- In records and information disclosed outside of this institution, you will be assigned a unique code number for this study. The Principal Investigator will ensure that the key to the code will be kept in a locked file. The key to the code will be destroyed at the end of the research study.

5. How long will this institution be able to use or disclose your personal health information?

Your authorization for use and disclosure of your child's personal health information for this specific study does not expire. The data will be kept for 5 years after publication, as required by the American Psychological Association.

6. Access to your child's records

During your child's participation in this study, you and your child will not be able to access the results of the assessments or intervention. This will be done to prevent the knowledge of study results from affecting the reliability of the study. Your child's information will be available should an emergency arise that would require your treating physician to know this information to best treat your child. You will have access to the results and any study information that is part of that record when the study is over or earlier, if possible. The investigator is not required to release to you research information that is not specifically associated with your child.

7. Changing your mind

You do not have to sign this form. If you do not, you are choosing to not allow your child to join the research study. Your decision to not sign this permission will not affect any other treatment, health care, enrollment in health plans or eligibility for benefits to which your child is normally entitled.

You may withdraw from the study for any reason simply by explaining this to the Principal Investigator or a member of the study team. If you decide not to allow your child to participate, you are free to leave the study at any time without consequence.

You may also withdraw your permission for the use and disclosure of any of your protected health information for research, **but you must do so in writing** to the Principal Investigator at the address on the first page. Even if you withdraw your permission, the Principal Investigator for the research study may still use your protected health information that was collected prior to your written request if that

information is necessary to the study. If you withdraw your permission to use your protected health information that means you will also be withdrawn from the research study.

Who can I call about my rights as a research subject?

If you have questions regarding your child's participation in this research study or if you have any questions about your child's rights as a research subject don't hesitate to speak with the Principal Investigator listed on page one of this form. Concerning your child's rights as a research subject, you may also contact the [redacted] Institutional Review Board by calling [redacted]. You may also contact the [redacted] at [redacted].

A description of this clinical trial will be available on http://www.ClinicalTrials.gov, as required by U.S. Law. This Web site will not include information that can identify you. At most, the Web site will include a summary of the results. You can search this Web site at any time.

When you sign this form, you are agreeing to take part in this research study. This means that you have read the consent form, your questions have been answered, and you have decided to volunteer. Your signature also means that you are permitting this institution to use your personal health information collected about you for research purposes. You are also allowing this institution to disclose that personal health information to outside organizations or people involved with the operations of this study.

A **signed** copy of this consent form will be given to you.

Video and Photo Release:

Video footage with audio will be recorded at all sessions to ensure safety and adhesion to study protocols as well as to record the study. The footage is being taken to ensure the rights of your child as a research subject and for the researchers alike. By signing this form, you are giving authorization for the Principal Investigator and research team to record your child during participation of the study and to share the footage with the following items if emergencies arise or the research protocol is not properly followed:

- The [redacted] Institutional Review Boards (the committees charged with overseeing research on human subjects)

- The Office of Human Research Oversight (the office which monitors research studies)
- Authorized members of the [redacted] workforce who may need to access your information in the performance of their duties, for example: to provide treatment, to ensure integrity of the research, accounting or billing matters, etc.
- Representatives of the university committee and office that reviews and approves research studies, the Institutional Review Board (IRB) and Office for Protection of Research Subjects;
- Other representatives of the state and university responsible for ethical, regulatory, or financial oversight of research;
- Federal government regulatory agencies such as the Office of Human Research Protections in the Department of Health and Human Services;

We would like permission to use the video footage and additional still images of your child during the experiments for research and teaching purposes. If you decide to give permission, the full image of your child will be stored without personal identifiers associated with the files apart from the image or video itself. These videos and photos will not contain close up footage of your child's face. However, the face may still be visible and recognizable. These photos would be included in scholarly publications in print and/or electronic form, which will allow your child's images to be viewable by anyone reading the publications. Photos and videos may also be presented at meetings or conferences without any personal identifiers attached to the photos and videos other than the content itself.

_____ (initial) I agree to videotaping and storing of photographs of my child for future research
and teaching purposes.

_____ (initial) I DO NOT agree to videotaping and storing of photographs of my child for future
research and teaching purposes.

If you are interested in participating in future studies, go to http://danceneuroscience.kch.illinois.edu/ for more information.

C1. Signature Block for Parent Permission and Child Assent: I have read this document or it was read to me. I have been encouraged to ask questions and all my questions were answered.

Age of Child Subject: _______

Please designate the relationship to the child subject. ___________________

PLEASE NOTE: Only a parent or court-appointed guardian with a guardianship appointment that is documented to be within the scope of their authority: limited guardianship, plenary guardianship, guardian of the person, guardian of the estate, and temporary guardianship. (Health Care Surrogate Act, 755 ILCS 40; Juvenile Court Act of 1987, 705 ILCS 405/1-2)] may consent to a MINOR's participation in research.)

____

Printed Name of Parent or Court-Appointed Guardian of Child Subject

____

Signature of Parent or Court-Appointed Guardian of Child Subject

____

Date

Use of an impartial witness should be filled when the child subject's Parent or Court-Appointed Guardian speaks and understands English, but cannot read and write or is visually impaired. If this is not the case, skip and continue.

____

Printed Name of Impartial Witness

____

Signature of Impartial Witness

____

Date

C2. Signature Block for Researcher/Designee: I have explained the research to the participant and the participant's parent(s) or legal guardian(s). The research participant and parent(s)/guardian(s) were encouraged to ask questions and all questions were answered to their satisfaction. A signed copy of this form has been given to the participant's representative.

____
Printed Name of Person Obtaining Consent

____
Signature of Person Obtaining Consent

____
Date

C3. Assent Documentation

Written Assent (14-17 years of age)

Introduction
You are asked to participate in a research study conducted by Dr. [redacted] and associates from the [redacted]. You were selected as a possible participant in this study because you are a healthy child between the ages of 7 and 17 that has either been diagnosed with spastic/dystonic or dystonic cerebral palsy or are typically developing. Your participation in this research study is voluntary.

Why is this study being done?

We want to fill the need of a tool that can be used by doctors to properly identify dystonia and spasticity. We also want to test our system to see if it is able to help relieve symptoms of dystonia.

What will you do if you choose to be in this study?

If you decide to be part of this study, your parent or guardian will bring you to the [redacted]

We will ask your parents/guardians to give their permission for you to take part in this study, but even if your parents say "yes," you can still decide not to do this.

If you volunteer to participate in this study, the researcher will ask you to do the following:

You will come to initial testing where we will ask you to answer some questionnaires, do some movement tests, and push and pull on a robot while it takes measurements. These sessions will not go past an hour and 30 minutes. During the initial testing, we will put small sensors (they go on like stickers) on your arms and torso to see how your muscles are activating. If something unexpected happens and we don't finish all the testing in two days, we might ask you to come back again. Everyone participating in this study will do these initial tests. If you are currently partaking in physical therapy, you will continue with your normal physical therapy; otherwise, you will continue your normal exercise routine. After this, your participation in this study will look different depending on which group you are

assigned to. You will either be assigned to Group 1 or to Group 2 using randomization. Randomization is something like flipping a coin.

If you are in Group 1, you will only come to the testing sessions. If you're in Group 2, you will come back six times to play a game. Each of these sessions will take one hour. You will get the option to play a game with a virtual reality (VR) headset on. You'll be able to take it off whenever you want to if you choose. We will also be collecting force data from the device that is attached to the robot and data on muscle activity from the same sensors that we placed on you during the initial testing.

Everyone will be asked to come back again (both Groups 1 and 2) to do some of the same tests we did in the beginning. After that testing (post-testing session), you will be asked to come back again to do the same thing about three weeks later (one-month post-testing session).

None of the measurements or activities should hurt and the activity will not be difficult. All sessions will be done individually, so no other kids will be there with you, but we will ask your parent/guardian to stay while you do any testing.

How long will I be in the research study?

Participation in the study will be over 6 to 7 weeks.

Are there any potential risks or discomforts that I can expect from this study?

There may be unexpected or unanticipated problems that come up during your participation in this study. Some risks may be currently unknown or unforeseeable. Most of the risks are listed below, but they will vary from person to person. You may discuss the listed risks with the research team and/or your regular physician if you would like. Risks related to the intervention and device we are studying include:

- Discomfort when using the VR headset that may cause movement sickness
- Physical discomfort when using the VR headset which may be caused by the weight of the headset
    - The headset is not required for participation in the study and may be removed at any time
- Muscle soreness due to repetitive movements
    - `You may take breaks and drink water as desired to alleviate this discomfort
- Irritation from the tape we use for the muscle sensors
    - We will have lotion available for you to help in taking the tape off
- Slight chance of seizures when using a virtual reality heasdset or playing a video game on a screen of any kind.
    - In the event of a seizure, an ambulance will be called.

Are there any potential benefits if I participate?

You may not directly benefit from your participation in the research. The results of the experiment may test if the intervention is beneficial for people diagnosed with dystonic cerebral palsy. It may also become possible to understand dystonia better. This new knowledge will benefit future patients and medical investigators.

Will I receive any payment if I participate in this study?

You will receive [redacted] per time you come in. The money will be given to you at the end of the study in the form of a gift card. If, for some reason, the session cannot be completed one day, you will be asked to come back and repeat the testing and will receive additional payment of [redacted] for each repeated visit. If you choose to withdraw from the study early for any reason, the amount will be adjusted at a rate of [redacted] per session. You will receive no more than [redacted].

Will information about me and my participation be kept confidential?

Any information that is obtained in connection with this study and that identify you will remain confidential. It will be disclosed only with your permission or as required by law.
Confidentiality will be maintained by storing any data on encrypted and password-protected computers. Any paperwork we have you fill out will be kept in a safe place that requires passage of multiple locks. When publishing the results of the study or presenting the gathered data at meetings, **no** identifying information (i.e. your name), will be associated with the data.

Withdrawal of participation by the investigator

The investigator may withdraw you from participating in this research if circumstances arise which warrant doing so. If you choose not to participate as is required, you may have to drop out, even if you would like to continue. The investigator will make the decision and let you know if it is not possible for you to continue.

What are my rights if I take part in this study?

You can choose whether or not you want to be in this study. If you volunteer to be in this study, you may leave the study at any time without consequences of any kind. You are not waiving any of your legal rights if you choose to be in this research study. You may refuse to answer any questions that you do not want to answer and still remain in the study.

Who can answer questions I might have about this study?

If you have any questions, comments or concerns about the research, you can talk to the one of the researchers or contact Dr. [redacted] at [redacted]

If you have questions about your rights as a research subject, or you have concerns or suggestions and you want to talk to someone other than the researchers, you may contact the [redacted] Institutional Review Board by calling [redacted]. You may also contact the [redacted] Office for the Protection of Research Subjects at [redacted].

Video and Photo Release:

Video footage with audio will be recorded at all sessions for safety. The footage is being taken to ensure your rights as a research subject and for the researchers alike. By signing this form, you are allowing the Principal Investigator and research team to record you during participation of the study and to share the footage with necessary parties if emergencies come up or the research protocol is not properly followed.

We would also like your additional and **optional** permission to use the video footage and to take pictures of you during the experiments for **research and teaching purposes**. These pictures and videos will not be close up images of your face and will not show your name anywhere. Your face may still be visible and recognizable in these pictures. These pictures or videos will only be used for educational purposes. If you decide to give permission, the full image of you will be stored without personal identifiers (i.e. your name) associated with the files apart from the image or video itself. These photos would be included in scholarly publications in print and/or electronic form, which will allow pictures of you to be viewable by anyone reading the publications. Photos and videos may also be presented at meetings or conferences without any personal identifiers attached to the photos and videos other than the content itself.

_____ (initial) I agree to videotaping and storing of photographs of me for future research and teaching purposes.

_____ (initial) I DO NOT agree to videotaping and storing of photographs of me for future research and teaching purposes.

____

Printed Name of Child Subject

Signature of Child Subject (Assent)

Date

Verbal Assent Documentation (7-13 years of age)

Hi my name is ***XX***. If you have any questions about what I am telling you, you can ask me at any time.

I want to tell you about a research study we are doing. In this study, we want to find out more about how the brain tells muscles to move so that doctors can diagnose their patients well and also to see if our system is able to change the way people can move.

You are being asked to be in this study because you are a healthy child between the ages of 7 and 17 and have or have not been diagnosed with dystonic cerebral palsy.

We will do something like flip a coin to decide if you will also play a game or not. If it is okay with you, I will ask you to come in a few different times to take some measurements, and if you end up in the group that plays a game, I will ask you to come in and play a computer game too. Each time we take measurements, it will take less than one hour and 30 minutes. We will ask you to use a robot and push and pull on it a few times. We will also measure how your muscles are working while you do this. This should not hurt at all. We will use sensors that are like stickers and we will put a few of them on your arms and back. We will videotape you for safety reasons.

If you are in the group that plays the game, you will come in six separate times where you will still push and pull on the robot to play the game and you will still have your muscles being measured. This will last about one hour. None of the measurements or activities should hurt and the activity will not be too difficult. We will videotape you for safety reasons.

Your ***parents/people taking care of you*** say it is okay for you to be in this study. If you have questions for me or for your ***parents/people who care for you***, you can ask them now or later.

If you get too tired or if this seems too silly just let me know. If you want to stop at any time, just tell me and we will stop. You do not have to be in this study. It is totally up to you. You can say yes now and still change your mind later. All you have to do is tell me. No one will be mad at you if you change your mind.

Do you understand what I am saying and are you willing to come to the testing sessions and use the robot, have your muscles measured, and be videotaped? Also, if you are in the group that plays a game, are you willing to come and play the game?

We would also like to take pictures and videos of you during the study that we can share with other researchers if you say that it is okay. Your face might be in the pictures but they won't be close up and we won't tell anyone it's you or what your name is. Are you willing to be photographed and videotaped and have these pictures and videos shared with other researchers?

End of verbal script.

Please mark as appropriate:

☐ **The research was explained to the participant in age-appropriate terms**

and the subject verbally agreed to take part in the study.
☐ Optional photo/video usage response:
☐ Yes ☐ No

☐ The participant declined to take part in the study. The participant declined for the following reason(s):

Check which applies below:

☐ The child/participant is capable of understanding the study
☐ The child/participant is not capable of understanding the study

An assent discussion was not initiated with the participant for the following reason(s):

☐ Subject is under 7 years of age.
☐ Subject is incapacitated.
☐ Subject refused to take part in the discussion.
☐ Other:
